# Anatomical projections of the dorsomedial hypothalamus to the periaqueductal grey and their role in thermoregulation: a cautionary note

**DOI:** 10.14814/phy2.13807

**Published:** 2018-07-25

**Authors:** Kathy C. G. de Git, Diana C. van Tuijl, Mieneke C. M. Luijendijk, Inge G. Wolterink‐Donselaar, Alexander Ghanem, Karl‐Klaus Conzelmann, Roger A. H. Adan

**Affiliations:** ^1^ Brain Center Rudolf Magnus Department of Translational Neuroscience University Medical Center Utrecht Utrecht University Utrecht The Netherlands; ^2^ Virology Faculty of Medicine Max von Pettenkofer Institute & Gene Center LMU München Munich Germany

**Keywords:** Brown adipose tissue, dorsomedial hypothalamus, periaqueductal grey, thermogenesis

## Abstract

The DMH is known to regulate brown adipose tissue (BAT) thermogenesis via projections to sympathetic premotor neurons in the raphe pallidus, but there is evidence that the periaqueductal gray (PAG) is also an important relay in the descending pathways regulating thermogenesis. The anatomical projections from the DMH to the PAG subdivisions and their function are largely elusive, and may differ per anterior–posterior level from bregma. We here aimed to investigate the anatomical projections from the DMH to the PAG along the entire anterior–posterior axis of the PAG, and to study the role of these projections in thermogenesis in Wistar rats. Anterograde channel rhodopsin viral tracing showed that the DMH projects especially to the dorsal and lateral PAG. Retrograde rabies viral tracing confirmed this, but also indicated that the PAG receives a diffuse input from the DMH and adjacent hypothalamic subregions. We aimed to study the role of the identified DMH to PAG projections in thermogenesis in conscious rats by specifically activating them using a combination of canine adenovirus‐2 (CAV2Cre) and Cre‐dependent designer receptor exclusively activated by designer drugs (DREADD) technology. Chemogenetic activation of DMH to PAG projections increased BAT temperature and core body temperature, but we cannot exclude the possibility that at least some thermogenic effects were mediated by adjacent hypothalamic subregions due to difficulties in specifically targeting the DMH and distinct subdivisions of the PAG because of diffuse virus expression. To conclude, our study shows the complexity of the anatomical and functional connection between the hypothalamus and the PAG, and some technical challenges in studying their connection.

## Introduction

Humans have evolved efficient physiological mechanisms that promote the acquirement and defense of energy stores in white adipose tissue (Seale and Lazar 2009). Pathological accumulation of energy stores results in obesity, a condition that is difficult to counteract with dieting alone, as decreased caloric intake is followed by physiological counter regulatory mechanisms that defend acquired energy stores (Seale and Lazar 2009; Maclean et al. 2011). A reduction in thermogenesis during dieting is one such a mechanism (Rosenbaum and Leibel 2010). Therefore, it is important to understand how thermogenesis is regulated.

Nonshivering or adaptive thermogenesis is controlled via brown adipose tissue (BAT), which has the specific metabolic function to dissipate energy in the form of heat (Dimicco and Zaretsky 2007; Clapham 2012; Heeren and Munzberg 2013; Rezai‐Zadeh and Munzberg 2013; Morrison et al. 2014; Morrison 2016). BAT is an important thermoregulatory effector organ in rodents in various physiological conditions (Dimicco and Zaretsky 2007; Morrison 2016), and recent evidence acknowledged metabolically active BAT in adult humans (Nedergaard et al. 2007; Cypess et al. 2009; van Marken Lichtenbelt et al. 2009; Virtanen et al. 2009). BAT thermogenesis is governed by central pathways that control sympathetic innervation of BAT (Dimicco and Zaretsky 2007; Clapham 2012; Heeren and Munzberg 2013; Rezai‐Zadeh and Munzberg 2013; Morrison et al. 2014; Morrison 2016). The DMH is now recognized as one of the key players in the thermoregulatory circuit (Dimicco and Zaretsky 2007; Morrison et al. 2008; Clapham 2012; Heeren and Munzberg 2013; Rezai‐Zadeh et al. 2014), as disinhibition of DMH neurons was shown to elicit a marked and rapid increase in BAT sympathetic nerve activity (SNA) and BAT temperature (Zaretskaia et al. 2002; Cao et al. 2004) that preceded the increase in core body temperature (Zaretskaia et al. 2002; de Menezes et al. 2006). The DMH is proposed to exert its sympathetic control of BAT thermogenesis via projections to sympathetic premotor neurons in the raphe pallidus (Chen et al. 2002; Cao et al. 2004; Rathner and Morrison 2006; Dimicco and Zaretsky 2007; Morrison et al. 2008; Clapham 2012; Heeren and Munzberg 2013), but there is evidence that the periaqueductal grey (PAG) is also an important relay in the descending pathways mediating thermogenesis (Dimicco and Zaretsky 2007; Morrison et al. 2008; de Menezes et al. 2006; Rathner and Morrison 2006 but see Nakamura and Morrison 2006).

The PAG is a relatively long midbrain region that can be divided into dorsomedial (dmPAG), dorsolateral (dlPAG), lateral (lPAG), and ventrolateral (vlPAG) subdivisions, which differ with respect to their functional properties and anatomical connections (Dampney et al. 2013). At least some of these subdivisions may have an opposite function in rostral versus caudal PAG extensions (de Menezes et al. 2006). With regard to thermogenesis, it seems that neuronal activity in the rostral vPAG functions to inhibit BAT SNA and BAT temperature (Rathner and Morrison 2006), whereas neuronal activity in the caudal (v)lPAG functions to increase BAT temperature (Chen et al. 2002). Neuronal activity in the caudal l/dlPAG (de Menezes et al. 2006, 2009) but not caudal (v)lPAG was shown to increase core body temperature (Chen et al. 2002). In accordance, the increase in BAT SNA and core body temperature arising from chemical disinhibition of DMH neurons was substantially reduced by chemical activation of the rostral vPAG (Rathner and Morrison 2006) and chemical inhibition of the caudal l/dlPAG (de Menezes et al. 2006), respectively. This suggests that the DMH increases thermogenesis through a combination of an inhibition of BAT sympathoinhibitory neurons in the rostral vPAG, and a facilitation of BAT sympathoexcitatory neurons in the caudal l/dlPAG. It should be noted that the role of the different PAG subdivisions in thermoregulation is difficult to interpret, as the subdivisions were assessed in independent studies with different experimental settings with, for example, the use of anesthesia (Chen et al. 2002; Rathner and Morrison 2006) or not (de Menezes et al. 2006, 2009), and the anatomical resolution of the microinjection techniques used is limited (Dimicco and Zaretsky 2007), which may result in misinterpretation of the targeted PAG subdivision.

The above‐mentioned functional connection between the DMH and PAG was only studied at very specific subdivisions in the PAG, that is, the vPAG at −5.3 to −5.6 mm from bregma, and the l/dlPAG at −7.64 to −8.30 from bregma. Clues for a potential role of other PAG subdivisions as a relay in the descending pathways from the DMH in mediating thermogenesis cannot be derived from previous anterograde tracing studies from the DMH to PAG, as these studies did not specify the anterior–posterior level at which PAG projections were observed (Lee et al. 2013; Papp and Palkovits 2014) or only described the PAG projections at a limited number of anterior–posterior levels from bregma (ter Horst and Luiten 1986; Thompson et al. 1996). The limited available evidence suggests that the projections from the DMH may vary largely per anterior–posterior level in the PAG subdivisions (ter Horst and Luiten 1986; Thompson et al. 1996).

We here aimed to further investigate the anatomical and functional projection from the DMH to the PAG. We started with an anterograde tracing study to identify which subdivisions and anterior–posterior levels of the PAG receive projections from the DMH. We then performed a retrograde tracing study in order to confirm the identified projections. Finally, we aimed to compare the role of four of the identified DMH to PAG projections in thermoregulation by the combined use of a canine adenovirus‐2 (CAV2Cre) and Cre‐dependent designer receptor exclusively activated by designer drugs (DREADD) technology, a method that allows for the specific activation of neural pathways (Hnasko et al. 2006; Boender et al. 2014; Boekhoudt et al. 2016). CAV2Cre was injected into the PAG, where it infects nerve terminals and retrogradely delivers Cre in the DMH, which subsequently enables the expression of the adeno‐associated virus (AAV) containing DREADD hM_3_D(G_q_) that was injected into the DMH.

## Methods

### Animals

Adult male Wistar rats (Charles‐River, Germany) were used, weighing ~300 g at the time of surgery. Rats were group housed in experiments 1 and 2, and individually housed in experiment 3 in a controlled environment under a normal light/dark cycle (lights on between 0700 and 1900 h). Rats had ad libitum access to standard chow (Special Diet Service, UK) and tap water, unless stated otherwise. All experiments were performed in accordance with Dutch laws (Wet op de Dierproeven, 1996) and European regulations (Guideline 86/609/EEC), and were approved by the Animal Ethics Committee of Utrecht University.

### Experiment 1: anterograde tracing from DMH to PAG

#### Surgery

Prior to surgery, rats were anaesthetized by intramuscular fentanyl/fluanisone (0.315 mg/kg fentanyl, 10 mg/kg fluanisone, Hypnorm, Janssen Pharmaceutica, Belgium). Xylocaine was sprayed on the skull to provide local anesthesia (Lidocaine 100 mg/mL, AstraZeneca BV, the Netherlands). All rats received three daily perisurgical injections of carprofen (5 mg/kg, s.c. Carporal, AST Farma BV, the Netherlands), starting at the day of surgery. Rats (*n* = 3) were unilaterally injected with 0.3 *μ*L of AAV‐hSyn‐ChR‐YFP (4.8*10^12^ genomic copies/mL; UNC vector core) in the DMH (from bregma: anterior–posterior (AP): −2.30 mm, mediolateral (ML): +1.40 mm, dorsoventral (DV): −9.30 mm, at an angle of 5°), using a stereotactic apparatus and a microliter infusion system. Virus was injected through 34G needles, which were connected to a Hamilton microliter syringe with polyethylene tubing. By using a microinfusion pump, the injection speed of all injections was set at 0.1 *μ*L/min. Following infusion, the needles were left in place for 10 min to prevent backflow. Prior to these surgeries, we performed pilot experiments to compare injections between the microliter infusion system and the nanojet (where glass capillaries are used). As no differences were observed in the targeting of the injections between the two systems, we decided to use the microliter infusion system for all our experiments.

#### Tissue preparation

In order to allow for sufficient virus expression, rats were sacrificed 3 weeks after surgery. Rats were given a lethal dose of sodium pentobarbital (200 mg/mL, Euthanimal, Alfasan BV, The Netherlands), and were transcardially perfused with 0.9% NaCl followed by 4% paraformaldehyde (PFA) in phosphate‐buffered saline (PBS). Brains were excised and kept in 4% PFA for 24 h, and were subsequently saturated with 30% sucrose in PBS with 0.01% NaN_3_. Brains were snap frozen in isopentane between −60°C and −40°C, and sliced into 40 *μ*m sections using a cryostate (Leica, Germany). Tissue was collected in six series in cryoprotectant (25% glycerol; 25% ethylene‐glycol in PBS) and stored at −20°C.

#### Immunohistochemistry

Two series of brain slices were washed in PBS and subsequently blocked and permeabilized in blocking solution (PBS containing 10% fetal calf serum and 1% triton X‐100) for 2 h. Subsequently, slices were incubated overnight at 4°C with primary chicken anti‐GFP antibody (1:500, Abcam, UK) in blocking solution. After washing in PBS, brain slices were incubated with Alexa‐488 labeled secondary goat anti‐chicken antibody in blocking solution for 2 h. After washing in PBS, slices were mounted on SuperFrost glasses (VWR, Leuven) and covered with FluorSave (Milipore).

#### Histological analysis

Immunofluorescent slices were photographed and digitized using a Zeiss Axioskop 2 epifluorescent microscope (Zeiss, Germany). Slices were matched to the stereotaxic brain atlas from Paxinos and Watson (1998; fourth edition), using the fornix, mammillothalamic tract, and optic tract as landmarks for the DMH, and the fourth ventricle and overall shape of the PAG for the PAG. The injection site of AAV‐hSyn‐ChR‐YFP in the DMH was determined by the expression of cell bodies with GFP immunoreactivity. The relative abundance of GFP‐labeled fibers was evaluated in different subdivisions of the PAG from bregma −4.80 till 8.80 mm by the following grading: absence of labeled fibers (−), very low (±), low (+), moderate (++), and high (+++). For each rat, one brain slice per bregma was evaluated.

### Experiment 2: retrograde tracing from PAG to DMH

#### Surgery

A second group of rats (*n* = 10) underwent surgery under identical procedures as described for experiment 1, but were unilaterally injected with 0.3 *μ*L of a mixture of rabies SAD DG mCherry (SAD_G) (Wickersham et al. 2007; Zhang et al. 2011; Ghanem and Conzelmann 2016) and AAV‐hSyn‐YFP (UNC vector core) (final concentration in mixture: 2.33*10^8^ and 1.00*10^12^ genomic copies/mL, respectively) in the PAG. Rats were randomly divided into five groups of two and injected at five different coordinates in the PAG, respectively (from bregma: rat 7 and 8: AP −5.30 mm; ML +1.40 mm/<10°; DV −6.70 mm; rat 9 and 10: AP −6.30 mm; ML +1.40 mm/<10°; DV −6.50 mm; rat 11 and 12: AP −5.30 mm; ML +1.40 mm/<10°; DV −6.20 mm; rat 13 and 14: AP −7.80 mm; ML +2.20 mm/<10°; DV −6.50 mm; rat 15 and 16: AP −7.00 mm; ML +1.40 mm/<10°; DV −5.70 mm.)

#### Tissue preparation

Rats were sacrificed 1 week after surgery via identical procedures as described for experiment 1.

#### Immunohistochemistry

Two series of brain slices were stained via identical procedures as described for experiment 1, but slices were incubated with primary chicken anti‐GFP (1:500, Abcam, UK) and rabbit anti‐dsRed (1:500, Clontech) antibodies, and secondary Alexa‐488‐labeled goat anti‐chicken (1:500, Abcam, UK) and Alexa‐568‐labeled goat anti‐rabbit (1:500, Abcam, UK) antibodies.

#### Histological analysis

Immunofluorescent slices were photographed and digitized using a Zeiss Axioskop 2 epifluorescent microscope (Zeiss, Germany). Slices were matched to the stereotaxic brain atlas from Paxinos and Watson (1998; fourth edition), using the fornix, mammillothalamic tract, and optic tract as landmarks for the hypothalamus, and the fourth ventricle and overall shape of the PAG for the PAG. The PAG injection site of rabies was determined by the expression of cell bodies with GFP immunoreactivity. One rat showed no virus expression in the PAG, and was therefore excluded from the following analyses. The number of inputs in the hypothalamus was evaluated by the expression of cell bodies with mCherry immunoreactivity. Hypothalamic inputs were systematically counted at the six sections between −2.30 and −3.60 mm from bregma defined by the rat brain atlas Paxinos and Watson (1998; fourth edition). Sections of the rat brain atlas were made transparent in Photoshop (Adobe Photoshop CC 2015, Adobe Systems Software Ireland Ltd), and overlays were made with the immunohistochemical pictures. For each rat, one picture was used per section, resulting in six pictures per rat in the hypothalamus. Images were loaded in Image J (version 1.50b, National Institutes of Health, USA) and the number of inputs was counted blindly in both sites of defined subregions in the hypothalamus.

### Experiment 3: functional connection between the DMH and PAG

#### Surgery

A third group of rats (*n* = 16) underwent surgery under identical procedures as described for experiment 1, but were bilaterally injected with 0.3 *μ*L of AAV‐hSyn‐DIO‐hM_3_D(G_q_)‐mCherry (3.8*10^12^ genomic copies/mL; UNC vector core) in the DMH, and bilaterally injected with 0.3 *μ*L of a mixture of CAV2Cre (final concentration in mixture 1.8*10^12^ genomic copies/mL; IGMM, France) and AAV‐hSyn‐EYFP (final concentration in mixture 1.65*10^12^ genomic copies/mL; UNC vector core) in the PAG. Rats were randomly divided into four groups of four and injected at four different coordinates in the PAG, respectively (from bregma: group 1: AP −5.30 mm; ML ± 1.40 mm/<10°; DV −5.70 mm; group 2: AP −6.30 mm; ML ±1.40 mm/<10°; DV −5.70 mm; group 3: AP −7.30 mm; ML ± 1.40 mm/<10°; DV −5.70 mm; group 4: AP −8.30 mm; ML ±1.40 mm/<10°; DV −5.70 mm). In addition, an intra‐abdominal dual transmitter (TL11M3F40‐TT, Data Science International, USA) with leads to the portal vein in the liver and interscapular brown adipose tissue was implanted under fentanyl/fluanisone (0.315 mg/kg fentanyl, 10 mg/kg fluanisone, Hypnorm, Janssen Pharmaceutica, Belgium) and midazolam (2.5 mg/kg, i.p., Actavis, the Netherlands) anesthesia in order to record core body temperature, BAT temperature, and activity.

#### Effects of CNO on body temperature and activity

The home cage was placed on a receiver plate (DSI, USA) that received radiofrequency signals from the abdominal transmitter. The plate was connected to software (DSI, USA) that recorded core body temperature, BAT temperature, and locomotor activity every 2 min. Test sessions started 2.5 weeks after surgery to allow the CAV2Cre to infect the DMH and induce hM3D(Gq)‐mCherry expression. During a test session, body temperature and activity were measured in the absence of food to prevent confounding with food‐induced thermogenesis. Rats were food restricted at 9.00 h, injected with saline or CNO (i.p.) at 12.00 h according to a Latin square design, and food was returned at 17.00 h. Clozapine‐N‐oxide (CNO; kindly provided by Bryan Roth and NIMH) was dissolved to a concentration of 0.3 mg/kg/mL in sterile saline (0.9% NaCl). The interval between two test sessions was 4 days and treatments were reversed between the two sessions. Rats were habituated twice to this procedure with saline (i.p.) prior to testing.

The placement of the leads to the liver and interscapular brown adipose tissue was checked after sacrifice. All liver probes were placed correctly, but the BAT probe was misplaced in five rats because it was slipped out of the insoluble suture. These rats were therefore excluded from the BAT temperature analysis. In two rats (both belonging to the DMH miss group), the battery of the transmitter was prematurely emptied, resulting in missing telemetry data. As a consequence, all 11 rats in the DMH hit group were included in the liver temperature analysis, and seven of these rats were included in the BAT temperature analysis. In the DMH miss group, three of the five rats were included in the liver temperature analysis, and two rats were included in the BAT temperature analysis.

#### Tissue preparation

Rats were sacrificed 6 weeks after surgery via identical procedures as described for experiments 1 and 2.

#### Immunohistochemistry

One series of brain slices was stained via identical procedures as described for experiment 2.

#### Histological analysis

Immunofluorescent slices were photographed and digitized using a Zeiss Axioskop 2 epifluorescent microscope (Zeiss, Germany). The injection site of AAV‐hSyn‐DIO‐hM3D(Gq)‐mCherry was determined by the expression of cell bodies with mCherry immunoreactivity, and the injection site of CAV2Cre was determined by the expression of cell bodies with GFP immunoreactivity, resulting from the coinjected AAV‐hSyn‐EYFP virus.

#### Data analysis

Telemetry data were filtered for unreliable values (which likely resulted from electromagnetic interference with the environment): all data points with a nonphysiological temperature (i.e., <35 or >40°C; ~6% of the data) were removed. Telemetry data were analyzed using a two‐way repeated measures ANOVA with time (in minutes) and treatment as within‐subject variable. When appropriate, post hoc analyses were conducted using pairwise Bonferroni comparisons. Each parameter was tested for normality with the Kolmogorov–Smirnov test. Statistical analyses were conducted using SPSS 20.3 for Windows. The threshold for statistical significance was set at *P* < 0.05. Data are presented as mean ± SEM.

## Results

### Anterograde tracing study from DMH to PAG

**Figure 1:**
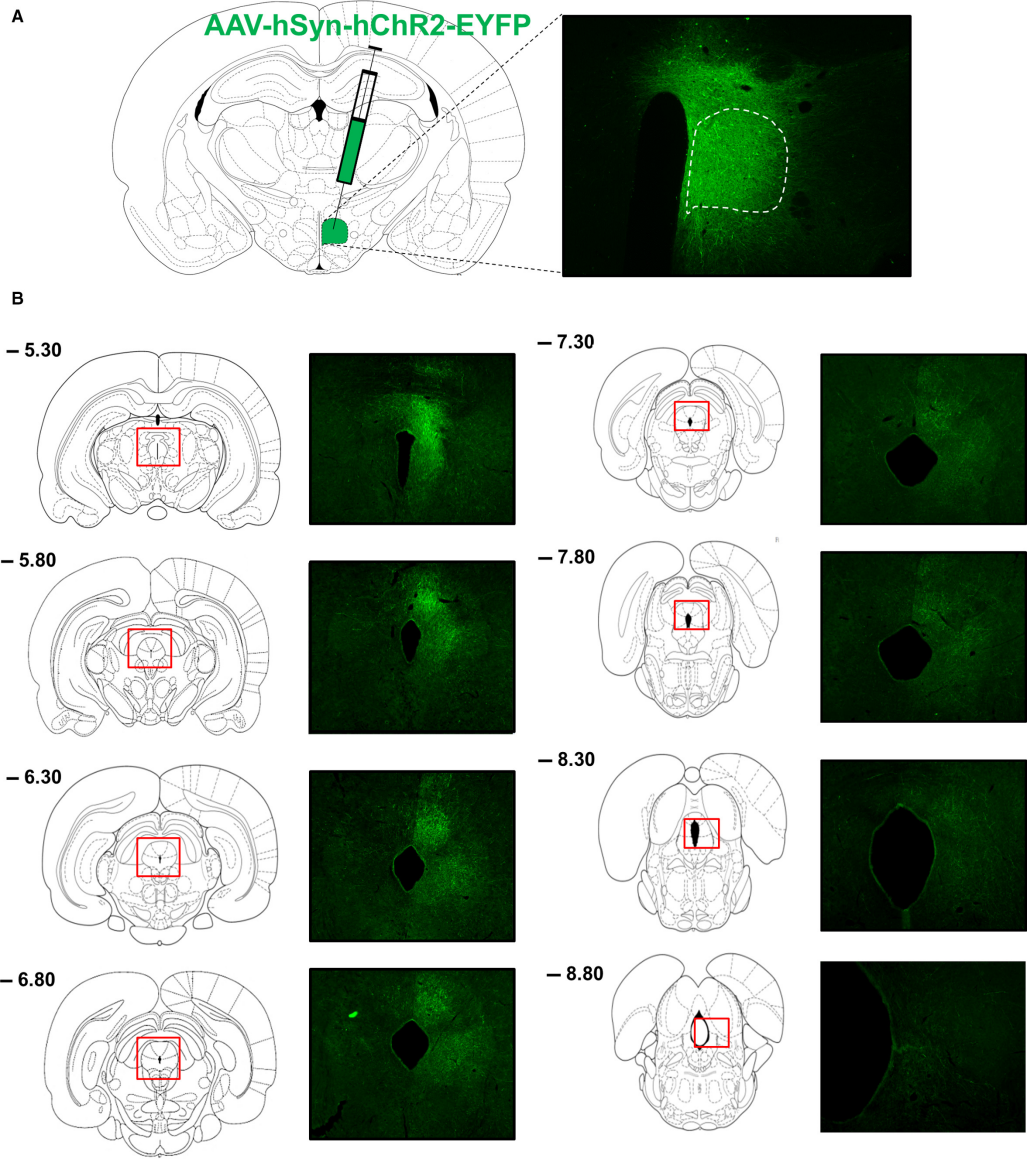
Anterograde tracing from DMH to PAG. (A) The anterograde tracer virus AAV‐hSyn‐hChR2‐EYFP was unilaterally injected into the DMH. GFP expression is shown for a successful injection in rat 2. The dotted outline shows the boundary of the DMH. GFP‐positive cell bodies were observed in the DMH, but not completely limited to this area. (B) Rostral to caudal GFP fiber distribution in subdivisions of the PAG. Distances from bregma (mm) are indicated at the left top.

**Figure 2:**
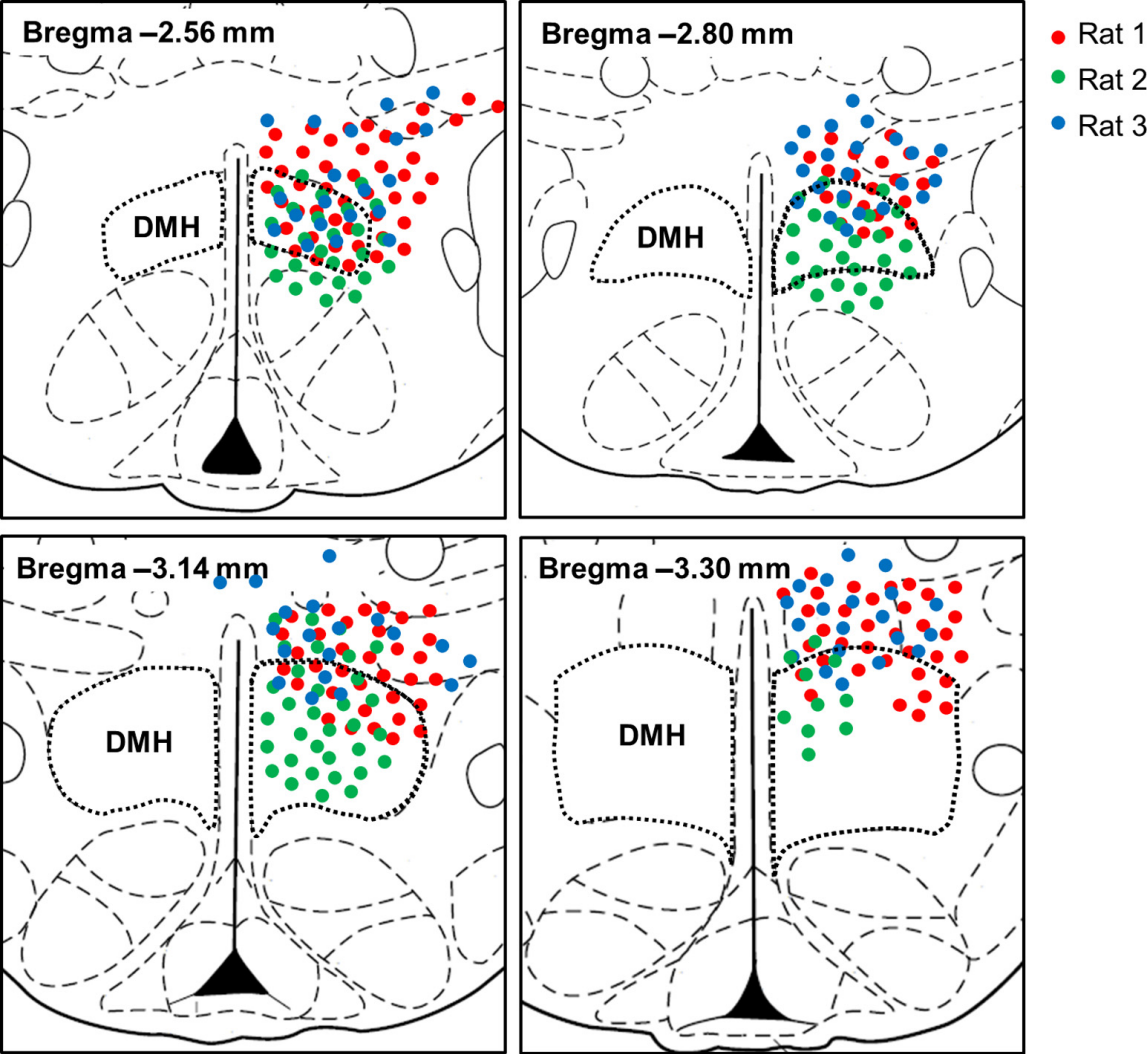
Mapping of AAV‐hSyn‐hChR2‐EYFP injection sites. Red, green, and blue circles indicate the expression of GFP‐positive cell bodies in rat 1, 2, and 3, respectively, in rostral to caudal levels of the rat hypothalamus. Distances from bregma (mm) are indicated at the left top. The anterior DMH was hit in all three rats. At more posterior bregma's, the dorsal DMH was hit in all rats, but rat 2 also showed GFP‐positive cell bodies in other subdivisions of the DMH. All rats showed some contamination of surrounding areas, especially the DHA.

To determine which anterior–posterior levels and subdivisions of the PAG receive input from the DMH, three rats were unilaterally injected with an anterograde tracer, AAV‐hSyn‐hChR2‐EYFP, in the DMH, and the relative abundance of GFP‐labeled fibers in the PAG was determined (Fig. 1A). The anterior DMH was hit in all three rats, but all rats showed some spread of expression in the surrounding areas (Fig. 2). One large injection (rat 2) labeled almost the entire DMH from the most anterior to the posterior part, and is therefore represented in detail (Fig. 1). The other two injection sites especially hit the dorsal DMH at the more posterior levels from bregma and also hit the DHA, an area that is often considered to form one thermogenic center together with the DMH (Heeren and Munzberg 2013; Rezai‐Zadeh and Munzberg 2013; Morrison et al. 2014; Rezai‐Zadeh et al. 2014; Morrison 2016) (Fig. 2).

The relative abundance of GFP‐labeled fibers was assessed in defined subdivisions of the PAG from its most anterior part (−4.80 mm from bregma) to its most posterior part (−8.80 mm from bregma). Rats showed similar patterns of fiber distribution in the PAG (Table 1). The projections were strongest in the most anterior and middle parts of the PAG and became weaker at the posterior parts (Table 1; Fig. 1B). Especially the dorsal and lateral PAG received projections from the DMH, but from the level of −8.00 mm from bregma onwards, the projections became weaker and more lateral.

**Table 1:** Relative densities of GFP‐positive fibers in the PAG originating from DMH neurons

	Rat 1	Rat 2	Rat 3	Average
PAG −4.80 mm				
dmPAG	+	+++	++	**++**
dlPAG	+	++	+	**+/++**
vlPAG	±	±	±	**±**
vmPAG	±	−	−	**−**
PAG −5.30 mm				
dmPAG	+	+++	+	**+/++**
dlPAG	+++	+++	++	**++/+++**
vlPAG	+	±	+	**+**
vmPAG	++	−	±	**+**
PAG −5.60 mm				
dmPAG	++	+++	++	**++**
dlPAG	+++	++	++	**++**
vlPAG	+++	++	±	**+/++**
vmPAG	++	±	−	**±**
PAG −5.80 mm				
dmPAG	++	+++	++	**++**
dlPAG	+++	+	±	**+**
lPAG	+++	++	+	**++**
vlPAG	++	−	±	**±**
vmPAG	++	−	−	**+**
PAG −6.30 mm				
dmPAG	+++	+++	++	**++/+++**
dlPAG	++	++	±	**+**
lPAG	+++	++	+	**++**
vlPAG	++	±	±	**+**
vmPAG	++	−	−	**±**
PAG −6.72 mm				
dmPAG	+++	+++	++	**++/+++**
dlPAG	+	+	±	**+**
lPAG	+++	++	++	**++**
vlPAG	+	−	±	**±**
vmPAG	++	−	−	**±**
PAG −6.80 mm				
dmPAG	+++	+++	++	**++/+++**
dlPAG	+	++	+	**+**
lPAG	+++	+++	++	**++**
vlPAG	++	±	±	**+**
vmPAG	++	−	−	**±**
PAG −7.30 mm				
dmPAG	+++	++	++	**++**
dlPAG	++	+	+	**+/++**
lPAG	+++	+	++	**++**
vlPAG	++	±	±	**+**
vmPAG	+	−	−	**±**
PAG −7.80 mm				
dmPAG	+++	++	+++	**++/+++**
dlPAG	++	+	+	**+**
lPAG	+++	++	+++	**++/+++**
vlPAG	+++	−	±	**+**
vmPAG	++	−	+	**+**
PAG −8.00 mm				
dmPAG	+	+	+	**+**
dlPAG	±	+	±	**±**
lPAG	+++	++	+	**++**
vlPAG	++	±	+	**+**
vmPAG	++	−	−	**+**
PAG −8.30 mm				
dmPAG	±	+	+	**+**
dlPAG	±	+	±	**±**
lPAG	++	+	+	**+**
vlPAG	++	+	+	**+**
vmPAG	+	−	±	**±**
PAG −8.80 mm				
dmPAG	±	−	±	**±**
lPAG	++	+	+	**+**
vlPAG	++	+	+	**+**
vmPAG	−	−	±	**−**

Overview of the relative densities of GFP‐positive fibers in anterior–posterior subdivisions of the PAG in rats injected with AAV‐hSyn‐hChR2‐EYFP in the DMH. −, absence of labeled fibers; ±, very low; +, low; ++, moderate; and +++, high. Distances from bregma (mm) are indicated. dmPAG, dorsomedial PAG; dlPAG, dorsolateral PAG; lPAG, lateral PAG; vlPAG, ventrolateral PAG; vmPAG, ventromedial PAG. The average result for the three rats (in bold).

### Retrograde tracing study from PAG to DMH

**Figure 3:**
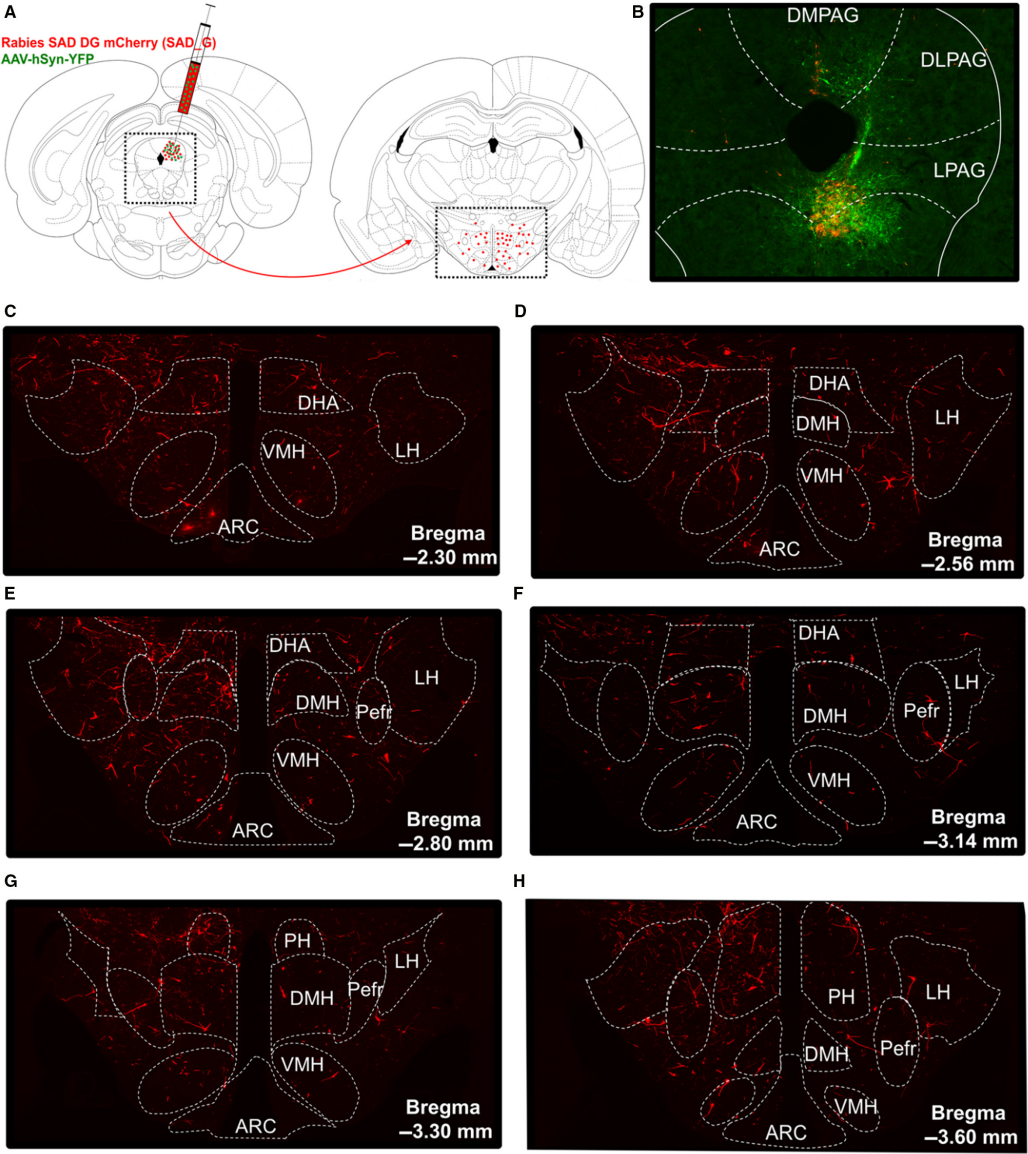
Retrograde tracing from PAG to hypothalamus. (A) Experimental design. Rabies SAD DG mCherry (SAD_G), a monosynaptic retrograde tracer virus, was unilaterally injected into the PAG together with an AAV‐hSyn‐YFP virus to visualize the injection site. The number of direct presynaptic inputs in defined subregions of the hypothalamus, including the DMH, was assessed. (B) Representative injection site, showing the needle track and most GFP‐positive cell bodies (green) in the ventral PAG. (C–H) Direct presynaptic inputs (red) in the anterior‐to‐posterior hypothalamus for the representative injection site in B. Distances from bregma (mm) are indicated, and the dotted outline shows the boundaries of hypothalamic subregions in which the number of presynaptic inputs was counted. ARC, arcuate nucleus; DHA, dorsohypothalamic area; DMH, dorsomedial hypothalamus, LH, lateral hypothalamus; Pefre, perifornical area; PH, posterior hypothalamus; VMH, ventromedial hypothalamus.

**Figure 4:**
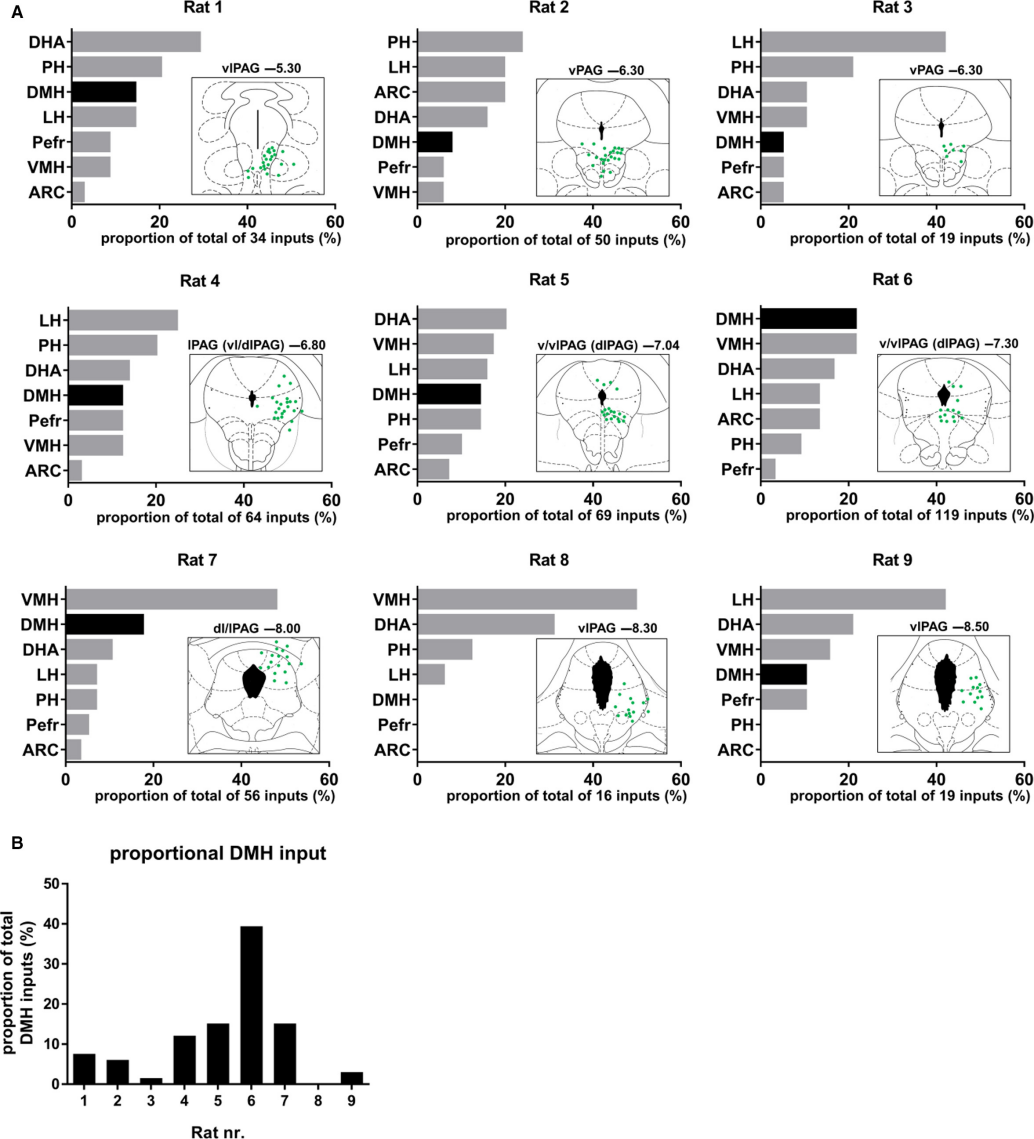
Hypothalamic monosynaptic inputs to the PAG. (A) Plots of the relative proportion of presynaptic inputs in defined subregions of the hypothalamus; and schematic overviews of the injection site for all individual rats that were injected with rabies SAD DG mCherry (SAD_G) into distinct subdivisions of the PAG. Hypothalamic subregions are ranked from highest to lowest number of inputs for each individual rat, and the DMH is highlighted. Rats are ranked from most anterior injection site (top left) to most posterior injection site (bottom right). The targeted subdivision of the PAG and distance from bregma (mm), as well as the total number of inputs are indicated for each injection site. (B) The normalized inputs in the DMH in each individual rat are normalized to the total number of inputs in the DMH of all rats.

In order to confirm the identified projections from the DMH to the dorsal and lateral PAG, we injected a retrograde rabies virus aimed at the d/lPAG at different anterior–posterior levels (Fig. 3A). Note that this recombinant rabies virus carries a rabies G coat, so that it infects all axon terminals near the injection site (Zhang et al. 2011; Ghanem and Conzelmann 2016). By using this retrograde tracer virus, we could assess the direct presynaptic inputs in the hypothalamus to the PAG. An AAV‐hSyn‐YFP virus was injected together with the rabies virus to visualize the injection site in the PAG. Analysis of the injection sites revealed that the vlPAG was hit in most rats instead of the targeted d/lPAG. One representative injection site is shown in Figure 3B, showing that especially the vPAG was hit, but also some virus expression was present in the dPAG. Figure 3C–H shows in detail the direct presynaptic inputs in defined subregions of the hypothalamus onto the PAG for the representative injection site, showing that the PAG receives widespread input from all hypothalamic subregions rather than specific input from the DMH. All injection sites resulted in widespread presynaptic inputs in the hypothalamus (Fig. 4A). The DMH was a relatively important hypothalamic input region in rats 6 and 7. Rat 7 was fully hit in the dlPAG and rat 6 also showed some GFP‐expressing neurons in the dlPAG, suggesting that the DMH is a relatively important input region of the dlPAG. Analysis of the number of inputs in the DMH in individual rats normalized to the number of inputs in the DMH in all rats revealed that rat 4, 5, 6, and 7 show relatively more inputs in the DMH compared with other rats (Fig. 4B). All four rats showed some GFP‐expressing neurons in the dlPAG, suggesting that especially the dlPAG receives prominent input from the DMH. Taken together, the rabies retrograde tracing supports the finding of projections from the DMH to especially the dlPAG in the ChR anterograde tracing study, but also shows that there is a diffuse projection from the hypothalamus to the PAG rather than specific input from the DMH.

### Role of the DMH to dlPAG projection in thermoregulation

**Figure 5:**
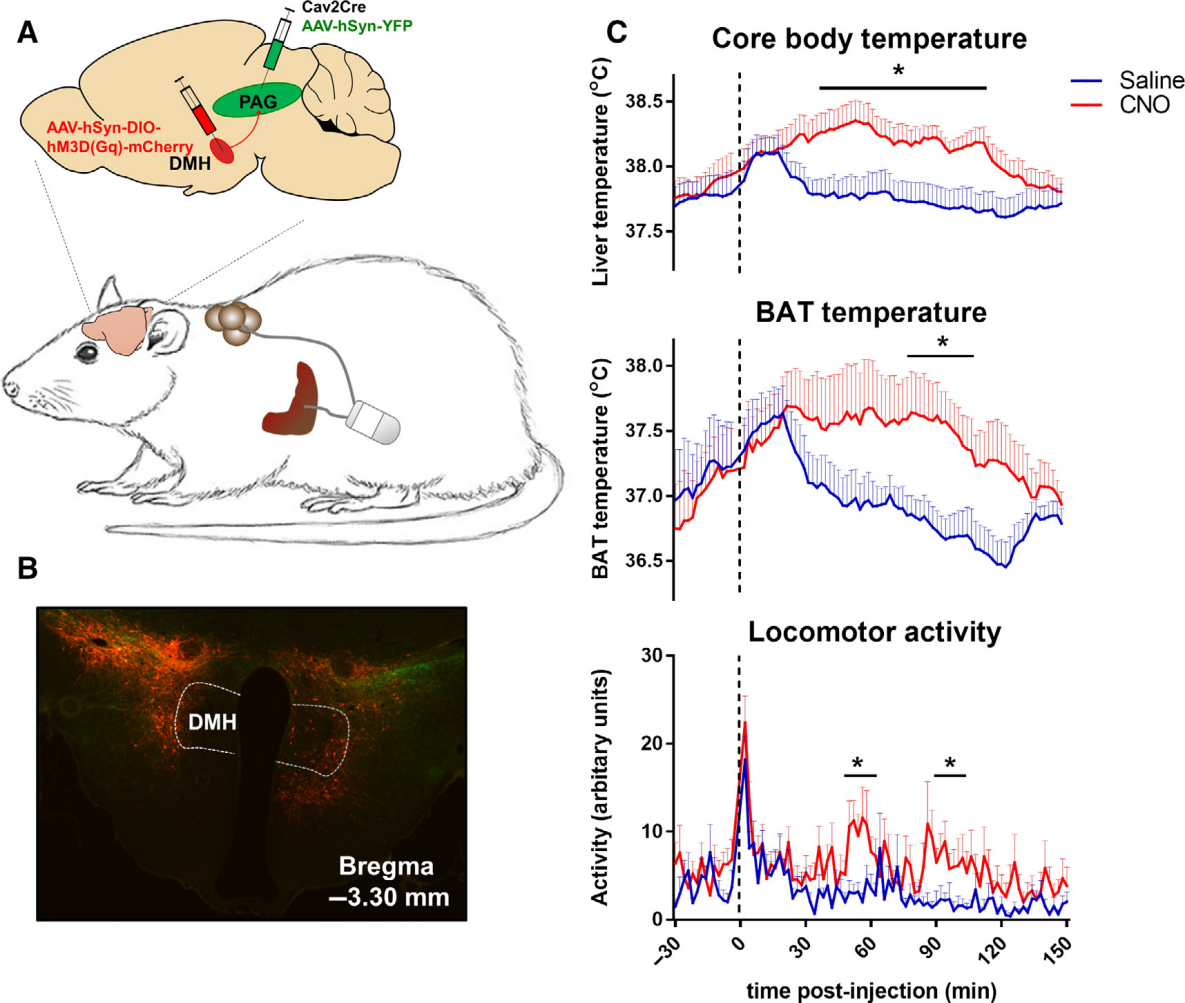
The effect of chemogenetic activation of DMH to PAG projections on thermoregulation. (A) Rats were implanted with an intraabdominal transmitter with one lead to the liver (to measure core body temperature) and one lead to brown adipose tissue (BAT). To selectively study the effect of dorsomedial hypothalamus (DMH) to periaqueductal grey (PAG) projections on thermoregulation, CAV2Cre was injected into the PAG and Cre‐dependent DREADD hM_3_D(G_q_) mCherry was injected into the DMH. An AAV‐hSyn‐YFP virus was injected together with CAV2Cre to visualize the injection site in the PAG. (B) Example injection site of DREADD hM_3_D(G_q_) in the DMH, showing hM3D(Gq)‐mCherry‐positive neurons in the DMH and surrounding areas. (C) CNO treatment in rats with hM3D(Gq)‐mCherry‐positive neurons in the DMH increased core body temperature, BAT temperature, and locomotor activity. Data are shown as mean ± SEM; *n* = 7–11.

To investigate whether the projections from the DMH to dlPAG control thermogenesis, we aimed to specifically activate this pathway by injecting CAV2Cre in the PAG and Cre‐dependent DREADD hM_3_D(G_q_) in the DMH (Fig. 5A). CAV2Cre infects neurons at terminals at the injection site and retrogradely delivers Cre in neurons that project to the area of injection, which subsequently enables the expression of Cre‐dependent DREADD hm_3_D(G_q_) in projection neurons (Hnasko et al. 2006; Boender et al. 2014; Boekhoudt et al. 2016). An AAV‐hSyn‐YFP virus was injected together with the CAV2Cre virus to visualize the injection site in the PAG.

We intended to specifically target the dlPAG at four different anterior–posterior levels (four rats per group), but our histology analysis revealed widespread virus expression in the PAG. Consequently, it was not possible to subgroup rats based on virus expression in the PAG, and results of all rats with virus expression in the PAG were combined. Analysis of DREADD hM_3_D(G_q_) expression (mCherry) in the hypothalamus indicated hM_3_D(G_q_)‐mCherry‐positive neurons in the DMH in 11 of the 16 injected rats, but hM_3_D(G_q_)‐mCherry expression was not restricted to the DMH (Fig. 5B). The DMH was not the main target, and it was impossible to define the predominant hypothalamic subregions with hM_3_D(G_q_)‐mCherry‐positive neurons due to diffuse mCherry staining around the injection site.

**Figure 6:**
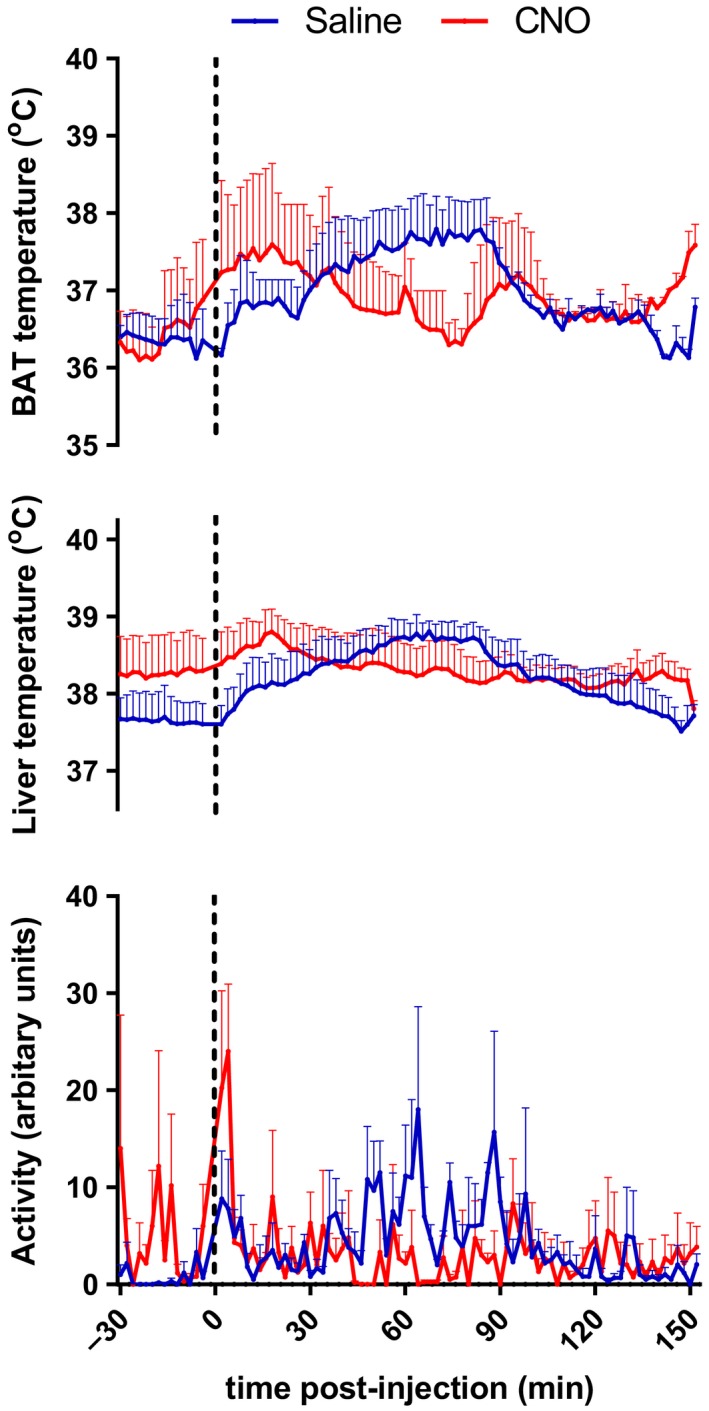
Thermoregulation in rats that show no hM3D(Gq)‐mCherry‐positive neurons in the DMH. CNO treatment in rats that showed no hM3D(Gq)‐mCherry‐positive neurons in the DMH did not have an effect on core body temperature (*F*_treatment(1,2)_ = 0.109, *P* = 0.773), BAT temperature (*F*_treatment(1,1)_ = 0.187, *P* = 0.740), and locomotor activity (*F*_treatment(1,2)_ = 3.409, *P* = 0.206). Data are shown as mean ± SEM;* n* = 2–3.

As a first step to assess the role of the DMH to PAG projection in thermogenesis, we combined results of all DMH hit rats and investigated the effect of chemogenetic activation with CNO (i.p., 0.3 mg/kg/mL) injections on thermoregulation. The temperature response to CNO differed significantly between DMH hit and DMH miss rats (core body and BAT temperature: *F*_treatment*time*group(74, 888/518)_ ≥ 2.824, *P* < 0.01). In DMH hit rats, CNO significantly increased core body temperature, starting after 30 min and returning to baseline within 150 min after injection (temperature response 1–150 min. after i.p., *F*_treatment*time(74, 740)_ = 1.259, *P* = 0.077; *F*_treatment(1,10)_ = 9.496, *P* = 0.012) (Fig. 5C). The rise in core body temperature may result from the significant increase in BAT thermogenesis, as the time course of the rise in BAT temperature was similar to that of core body temperature (*F*_treatment*time(74, 444)_ = 1.292, *P* = 0.063; *F*_treatment(1, 6)_ = 6.380, *P* = 0.045) (Fig. 5C). However, as CNO injections also increased locomotor activity (*F*_treatment*time(74, 740)_ = 1.253, *P* = 0.081; *F*_treatment(1, 10)_ = 15.595, *P* < 0.01) (Fig. 5C), the rise in core body temperature may not exclusively result from increased BAT thermogenesis. In rats that showed no hM_3_D(G_q_)‐mCherry‐positive neurons in the DMH, CNO treatment did not affect thermogenesis or locomotor activity (Fig. 6). Taken together, these findings support the literature showing that activation of DMH neurons projecting to the PAG increases thermogenesis (de Menezes et al. 2006; Rathner and Morrison 2006), but we cannot exclude that surrounding hypothalamic subregions contribute to this effect.

## Discussion

The PAG is a relatively long brain region with distinct subdivisions, representing longitudinal columns. The boundaries of these columns were previously established on the basis of anatomical connections, functional and chemical properties (Dampney et al. 2013). We here performed the first study of DMH projections to PAG subdivisions along the entire anterior–posterior axis of the PAG. Our study shows projections from the DMH to especially the d/lPAG, but also indicates that there is a diffuse projection from the hypothalamus to the PAG rather than specific input from the DMH. The predominant hypothalamic input areas of the PAG vary largely per injection site, but generally include the dorsohypothalamic area (DHA), posterior hypothalamus (PH), lateral hypothalamus (LH), and ventromedial hypothalamus (VMH), which are positioned directly adjacent the DMH. The points raised above illustrate the necessity for very specific virus injections in the relatively small DMH region of the hypothalamus and distinct subdivisions of the PAG to unravel their specific anatomical and functional connections, which appeared technically challenging.

### Anterograde tracing from DMH to PAG

In our anterograde tracing study from DMH to PAG, we aimed to specifically target the DMH, but observed some viral spread in the surrounding areas, especially the DHA. As the DHA has been shown to project to the PAG (Papp and Palkovits 2014), the projections we observed in the PAG were probably not specific for the DMH. Tracer spread to surrounding areas is a common problem in anterograde tracing studies of the DMH (ter Horst and Luiten 1986; Thompson et al. 1996; Lee et al. 2013). It is difficult to directly compare the extent of viral spread around the injection site with previous anterograde tracing studies, as the injection site was previously only shown for one anterior–posterior level from bregma and/or for only one or a few representative cases (ter Horst and Luiten 1986; Thompson et al. 1996; Lee et al. 2013).

Our finding that the anterior and middle parts of the PAG receive projections from the DMH in their dorsal and lateral subdivisions is roughly in accordance with the results of ter Horst and Luiten (1986) and Thompson et al. (1996), as far as could be determined at the limited number of anterior–posterior levels they presented. However, in the posterior PAG, ter Horst and Luiten (1986) and Thompson et al. (1996) showed relatively more projections in the vlPAG. One limitation of our tracing study is that we were not able to discriminate between passing fibers and terminal fields. It is likely that a substantial portion of the fibers we observed in the PAG are axons of passage with some terminal fields interspersed, as this was previously shown by studies that used tracers that enable discrimination between passing fibers and terminal labeling (ter Horst and Luiten 1986; Thompson et al. 1996; Papp and Palkovits 2014). Such studies showed most abundant axonal branching and terminal fields in the posterior vlPAG (ter Horst and Luiten 1986; Thompson et al. 1996). Therefore, one explanation for why we observed most fibers in the d/lPAG, whereas previous studies reported the most prominent innervation in the vlPAG (often at unknown anterior–posterior levels from bregma) (Thompson et al. 1996; Lee et al. 2013; Papp and Palkovits 2014), may result from visualizing fibers versus terminal fields, respectively. Alternatively, differences in the location of the injection site in the DMH or in the surrounding area to which the virus spread may explain differences in projection patterns in the PAG. In comparison with previous studies, the injections sites in our study were located in more anterior parts of the DMH (ter Horst and Luiten 1986; Thompson et al. 1996; Lee et al. 2013; Papp and Palkovits 2014).

### Retrograde tracing from PAG to DMH

As we were not able to discriminate between passing fibers and terminal ends in our anterograde tracing from the DMH to the PAG, we intended to confirm the identified projections by injecting a retrograde rabies virus in the dlPAG at different anterior–posterior levels. Because this tracer does not label the injection site, we coinjected an AAV‐GFP virus to visualize the injection site, which only provides an indication of the injection site as the viral spread may be different for the rabies versus AAV virus. Analysis of the estimated injection sites indicates that most rats were hit in the vlPAG instead of the targeted dlPAG, which complicates the ability to confirm the identified projections from the DMH to the dlPAG. Nevertheless, the four rats that showed relatively more inputs in the DMH compared with other rats, all showed some expression in the dlPAG around −6.80 mm till −8.00 mm from bregma, which supports the findings of the anterograde tracing study that especially the d/lPAG receives input from the DMH. The existence of anatomical connections between the DMH and dlPAG around −6.80 mm till −8.00 mm from bregma is supported by physiological data. Studies showing that the PAG is an important relay in the descending pathways mediating the thermogenic, cardiovascular, and locomotor response evoked by activation of the DMH, all targeted the dlPAG between −6.80 mm till −8.30 mm from bregma (da Silva et al. 2003; Yoshida et al. 2005; Da Silva et al. 2006; de Menezes et al. 2006; Villela et al. 2009), and found no effect of chemical inhibition of the vlPAG on DMH‐evoked responses (Villela et al. 2009).

One important finding of the rabies tracing is that the PAG receives a diffuse input from the hypothalamus, rather than a specific input from the DMH. This finding is supported by literature showing that other hypothalamic regions, including the LH, VMH, perifornical area (PeF), DHA, and PH, project to the PAG (Saper et al. 1976; Canteras et al. 1994; Thornhill and Halvorson 1994; Mota‐Ortiz et al. 2009; Papp and Palkovits 2014). We here show that the hypothalamic areas providing predominant input to the PAG vary largely per injection site, but generally include the DHA, PH, LH, and VMH. The size and cell density differ between the hypothalamic subregions that were assessed. These differences create evaluation problems of the relative importance of input from the DMH compared with other hypothalamic subregions. Therefore, comparison of the relative importance of the input from the distinct hypothalamic subregions needs caution. Despite this limitation, it is obvious that the predominant input areas vary largely per PAG subdivision, and that the DMH is generally not one of the major hypothalamic input regions.

### Role of the DMH to PAG projection in thermoregulation

Interestingly, the hypothalamic subregions that provided the predominant input to the PAG in the rabies tracing, that is, the DHA, PH, LH, and VMH, were all shown to mediate thermogenesis (Minokoshi et al. 1986; Holt et al. 1987; Amir 1990a,b; Halvorson et al. 1990; Kobayashi et al. 1999; Nakamura and Morrison 2006; Horiuchi et al. 2009; Morrison et al. 2012; Rezai‐Zadeh et al. 2014; Belanger‐Willoughby et al. 2016). As these subregions all lie in the immediate proximity of the DMH, it is critical to specifically hit the DMH in intervention studies that aim to unravel the role of the PAG in DMH‐evoked thermogenesis.

We here aimed to selectively activate neurons projecting from the DMH to distinct subdivisions of the PAG by the combined use of CAV2Cre and Cre‐dependent DREADD technology. In principle, this method allows more selective activation of DMH to PAG projections than the method employed by previous studies (da Silva et al. 2003; Yoshida et al. 2005; Da Silva et al. 2006; de Menezes et al. 2006; Rathner and Morrison 2006; Villela et al. 2009). In those studies, the DMH to PAG connection was modulated by chemical (dis)inhibition of the DMH and PAG via local injection of drugs, which does not allow for the specific activation (or disinhibition) of DMH neurons that project to the PAG. However, we failed to specifically hit the DMH and distinct subdivisions of the PAG because of diffuse virus expression. Explanations for the diffuse virus expression could be a too large injection volume or too high virus titer. Our injection volume of 300 nL is relatively large compared with the 20–100 nL that was injected in studies that chemically (dis)inhibited the DMH and PAG (Da Silva et al. 2006; de Menezes et al. 2006; Rathner and Morrison 2006; Villela et al. 2009), but is smaller compared with other studies that used Cre‐dependent DREADD technology (Boender et al. 2014; Boekhoudt et al. 2016). The titers we used for CAV2Cre and DREADD hM_3_D(G_q_) were relatively high compared with previous studies (Boender et al. 2014; Boekhoudt et al. 2016).

This is the first study that aimed to investigate the role of the DMH to PAG projection on core body and brown adipose tissue thermogenesis in conscious rats. Our findings suggest that the increase in core body temperature results mostly from the larger increase in BAT temperature, as the time course of the rise in both temperatures was similar. We found a small increase in locomotor activity that might contribute to the increase in core body temperature, but in our study locomotor activity is not likely to be an important contributor because of the delayed and unstable response pattern. Locomotor activity was previously also shown not to be causal to DMH‐evoked increases in core body temperature (de Menezes et al. 2006). It should be noted that reduced heat loss via the tail through vasoconstriction might also contribute to the CNO‐induced increase in core body temperature, as the tail is an important thermoregulatory effector organ in rodents, and both the DMH and PAG have been implicated in the regulation of tail blood flow (Chen et al. 2002; de Menezes et al. 2006; Dimicco and Zaretsky 2007; Rezai‐Zadeh and Munzberg 2013; Morrison 2016). Because of the diffuse virus injections, we cannot exclude the possibility that other hypothalamic subregions, that were hit in the DMH‐hit but not the DMH‐miss group, mediated the CNO effects on BAT and core body thermogenesis that were only observed in DMH hit rats. Importantly, the histology analysis showed that the DMH often contains less hM_3_d(G_q_)‐mCherry‐positive neurons compared with the surrounding hypothalamic subregions, which is in accordance with the rabies tracing result showing that the DMH is generally not one of the most important input areas for the PAG. Therefore, at least some of the thermogenic effects we observed were potentially mediated by hypothalamic subregions surrounding the DMH. Neurons in these subregions were shown to play a role in thermogenesis (Amir 1990a,b; Nakamura and Morrison 2006; Horiuchi et al. 2009; Rezai‐Zadeh et al. 2014) and project to the PAG (Saper et al. 1976; Canteras et al. 1994; Mota‐Ortiz et al. 2009; Papp and Palkovits 2014), but the function of their projection to the PAG has not been investigated yet.

## Conclusion

To conclude, we emphasize the importance of making specific, small virus injections into the DMH and PAG subdivisions, to study the precise anatomical and functional connection between them. This is a critical technical requirement because the PAG receives a diffuse input from the DMH and adjacent hypothalamic subregions, which were previously shown to play a role in thermoregulation as well (Minokoshi et al. 1986; Holt et al. 1987; Amir 1990a,b; Halvorson et al. 1990; Kobayashi et al. 1999; Nakamura and Morrison 2006; Horiuchi et al. 2009; Morrison et al. 2012; Rezai‐Zadeh et al. 2014; Belanger‐Willoughby et al. 2016). Performing (dual) specific virus injections appeared to be technically challenging in our experiments, resulting in an imprecise determination of the role of the DMH to PAG projections in thermoregulation. Thus, our study shows the complexity of the connection between the hypothalamus and the PAG, and demonstrates some of the limitations of (dual) viral vector technology.

## Conflict of Interest

The authors declare that no competing interests exist.

## References

[phy213807-bib-0001] Amir, S. 1990a Intra‐ventromedial hypothalamic injection of glutamate stimulates brown adipose tissue thermogenesis in the rat. Brain Res. 511:341–344. 1970749 10.1016/0006-8993(90)90181-a

[phy213807-bib-0002] Amir, S. 1990b Activation of brown adipose tissue thermogenesis by chemical stimulation of the posterior hypothalamus. Brain Res. 534:303–308. 1981483 10.1016/0006-8993(90)90145-2

[phy213807-bib-0003] Belanger‐Willoughby, N. , V. Linehan , and M. Hirasawa . 2016 Thermosensing mechanisms and their impairment by high‐fat diet in orexin neurons. Neuroscience 324:82–91. 26964685 10.1016/j.neuroscience.2016.03.003

[phy213807-bib-0004] Boekhoudt, L. , A. Omrani , M. C. Luijendijk , I. G. Wolterink‐Donselaar , E. C. Wijbrans , G. van der Plasse , et al. 2016 Chemogenetic activation of dopamine neurons in the ventral tegmental area, but not substantia nigra, induces hyperactivity in rats. Eur. Neuropsychopharmacol. 26:1784–1793. 27712862 10.1016/j.euroneuro.2016.09.003

[phy213807-bib-0005] Boender, A. J. , J. W. de Jong , L. Boekhoudt , M. C. Luijendijk , G. van der Plasse , and R. A. Adan . 2014 Combined use of the canine adenovirus‐2 and DREADD‐technology to activate specific neural pathways in vivo. PLoS ONE 9:e95392. 24736748 10.1371/journal.pone.0095392 PMC3988196

[phy213807-bib-0006] Canteras, N. S. , R. B. Simerly , and L. W. Swanson . 1994 Organization of projections from the ventromedial nucleus of the hypothalamus: a phaseolus vulgaris‐leucoagglutinin study in the rat. J. Comp. Neurol. 348:41–79. 7814684 10.1002/cne.903480103

[phy213807-bib-0007] Cao, W. H. , W. Fan , and S. F. Morrison . 2004 Medullary pathways mediating specific sympathetic responses to activation of dorsomedial hypothalamus. Neuroscience 126:229–240. 15145088 10.1016/j.neuroscience.2004.03.013

[phy213807-bib-0008] Chen, X. M. , M. Nishi , A. Taniguchi , K. Nagashima , M. Shibata , and K. Kanosue . 2002 The caudal periaqueductal gray participates in the activation of brown adipose tissue in rats. Neurosci. Lett. 331:17–20. 12359313 10.1016/s0304-3940(02)00757-7

[phy213807-bib-0009] Clapham, J. C. 2012 Central control of thermogenesis. Neuropharmacology 63:111–123. 22063719 10.1016/j.neuropharm.2011.10.014

[phy213807-bib-0010] Cypess, A. M. , S. Lehman , G. Williams , I. Tal , D. Rodman , A. B. Goldfine , et al. 2009 Identification and importance of brown adipose tissue in adult humans. N. Engl. J. Med. 360:1509–1517. 19357406 10.1056/NEJMoa0810780 PMC2859951

[phy213807-bib-0011] Da Silva Jr, L. G. , R. C. A. Menezes , D. C. Villela , and M. A. P. Fontes . 2006 Excitatory amino acid receptors in the periaqueductal gray mediate the cardiovascular response evoked by activation of dorsomedial hypothalamic neurons. Neuroscience 139:1129–1139. 16458440 10.1016/j.neuroscience.2005.12.041

[phy213807-bib-0012] Dampney, R. A. , T. M. Furlong , J. Horiuchi , and K. Iigaya . 2013 Role of dorsolateral periaqueductal grey in the coordinated regulation of cardiovascular and respiratory function. Auton. Neurosci. 175:17–25. 23336968 10.1016/j.autneu.2012.12.008

[phy213807-bib-0013] Dimicco, J. A. , and D. V. Zaretsky . 2007 The dorsomedial hypothalamus: a new player in thermoregulation. Am. J. Physiol. Regul. Integr. Comp. Physiol. 292:R47–R63. 16959861 10.1152/ajpregu.00498.2006

[phy213807-bib-0014] Ghanem, A. , and K. K. Conzelmann . 2016 G gene‐deficient single‐round rabies viruses for neuronal circuit analysis. Virus Res. 216:41–54. 26065596 10.1016/j.virusres.2015.05.023

[phy213807-bib-0015] Halvorson, I. , L. Gregor , and J. A. Thornhill . 1990 Brown adipose tissue thermogenesis is activated by electrical and chemical (L‐glutamate) stimulation of the ventromedial hypothalamic nucleus in cold‐acclimated rats. Brain Res. 522:76–82. 2224517 10.1016/0006-8993(90)91579-6

[phy213807-bib-0016] Heeren, J. , and H. Munzberg . 2013 Novel aspects of brown adipose tissue biology. Endocrinol. Metab. Clin. North Am. 42:89–107. 23391242 10.1016/j.ecl.2012.11.004 PMC3568264

[phy213807-bib-0017] Hnasko, T. S. , F. A. Perez , A. D. Scouras , E. A. Stoll , S. D. Gale , S. Luquet , et al. 2006 Cre recombinase‐mediated restoration of nigrostriatal dopamine in dopamine‐deficient mice reverses hypophagia and bradykinesia. Proc. Natl Acad. Sci. USA 103:8858–8863. 16723393 10.1073/pnas.0603081103 PMC1466546

[phy213807-bib-0018] Holt, S. J. , H. V. Wheal , and D. A. York . 1987 Hypothalamic control of brown adipose tissue in zucker lean and obese rats. effect of electrical stimulation of the ventromedial nucleus and other hypothalamic centres. Brain Res. 405:227–233. 3567603 10.1016/0006-8993(87)90292-7

[phy213807-bib-0019] Horiuchi, J. , L. M. McDowall , and R. A. Dampney . 2009 Vasomotor and respiratory responses evoked from the dorsolateral periaqueductal grey are mediated by the dorsomedial hypothalamus. J. Physiol. 587:5149–5162. 19752114 10.1113/jphysiol.2009.179739 PMC2790255

[phy213807-bib-0020] ter Horst, G. J. , and P. G. Luiten . 1986 The projections of the dorsomedial hypothalamic nucleus in the rat. Brain Res. Bull. 16:231–248. 3697791 10.1016/0361-9230(86)90038-9

[phy213807-bib-0021] Kobayashi, A. , T. Osaka , Y. Namba , S. Inoue , and S. Kimura . 1999 CGRP microinjection into the ventromedial or dorsomedial hypothalamic nucleus activates heat production. Brain Res. 827:176–184. 10320707 10.1016/s0006-8993(99)01333-5

[phy213807-bib-0022] Lee, S. J. , M. Kirigiti , S. R. Lindsley , A. Loche , C. J. Madden , S. F. Morrison , et al. 2013 Efferent projections of neuropeptide Y‐expressing neurons of the dorsomedial hypothalamus in chronic hyperphagic models. J. Comp. Neurol. 521:1891–1914. 23172177 10.1002/cne.23265 PMC3618613

[phy213807-bib-0023] Maclean, P. S. , A. Bergouignan , M. A. Cornier , and M. R. Jackman . 2011 Biology's response to dieting: the impetus for weight regain. Am. J. Physiol. Regul. Integr. Comp. Physiol. 301:R581–R600. 21677272 10.1152/ajpregu.00755.2010 PMC3174765

[phy213807-bib-0024] van Marken Lichtenbelt, W. D. , J. W. Vanhommerig , N. M. Smulders , J. M. Drossaerts , G. J. Kemerink , N. D. Bouvy , et al. 2009 Cold‐activated brown adipose tissue in healthy men. N. Engl. J. Med. 360:1500–1508. 19357405 10.1056/NEJMoa0808718

[phy213807-bib-0025] de Menezes, R. C. , D. V. Zaretsky , M. A. Fontes , and J. A. DiMicco . 2006 Microinjection of muscimol into caudal periaqueductal gray lowers body temperature and attenuates increases in temperature and activity evoked from the dorsomedial hypothalamus. Brain Res. 1092:129–137. 16677620 10.1016/j.brainres.2006.03.080

[phy213807-bib-0026] de Menezes, R. C. , D. V. Zaretsky , M. A. Fontes , and J. A. DiMicco . 2009 Cardiovascular and thermal responses evoked from the periaqueductal grey require neuronal activity in the hypothalamus. J. Physiol. 587:1201–1215. 19171660 10.1113/jphysiol.2008.161463 PMC2674992

[phy213807-bib-0027] Minokoshi, Y. , M. Saito , and T. Shimazu . 1986 Sympathetic denervation impairs responses of brown adipose tissue to VMH stimulation. Am. J. Physiol. 251:R1005–R1008. 3777207 10.1152/ajpregu.1986.251.5.R1005

[phy213807-bib-0028] Morrison, S. F. 2016 Central neural control of thermoregulation and brown adipose tissue. Auton. Neurosci. 196:14–24. 26924538 10.1016/j.autneu.2016.02.010 PMC4846468

[phy213807-bib-0029] Morrison, S. F. , K. Nakamura , and C. J. Madden . 2008 Central control of thermogenesis in mammals. Exp. Physiol. 93:773–797. 18469069 10.1113/expphysiol.2007.041848 PMC2496891

[phy213807-bib-0030] Morrison, S. F. , C. J. Madden , and D. Tupone . 2012 An orexinergic projection from perifornical hypothalamus to raphe pallidus increases rat brown adipose tissue thermogenesis. Adipocyte 1:116–120. 23538704 10.4161/adip.19736 PMC3607627

[phy213807-bib-0031] Morrison, S. F. , C. J. Madden , and D. Tupone . 2014 Central neural regulation of brown adipose tissue thermogenesis and energy expenditure. Cell Metab. 19:741–756. 24630813 10.1016/j.cmet.2014.02.007 PMC4016184

[phy213807-bib-0032] Mota‐Ortiz, S. R. , M. H. Sukikara , L. F. Felicio , and N. S. Canteras . 2009 Afferent connections to the rostrolateral part of the periaqueductal gray: a critical region influencing the motivation drive to hunt and forage. Neural. Plast. 2009:612698. 19325910 10.1155/2009/612698 PMC2657915

[phy213807-bib-0033] Nakamura, K. , and S. F. Morrison . 2006 Central efferent pathways mediating skin‐evoked sympathetic thermogenesis in brown adipose tissue. Am. J. Phyiol. Regul. Integr. Comp. Physiol. 292:R127–R136. 10.1152/ajpregu.00427.2006 PMC2441894 16931649

[phy213807-bib-0034] Nedergaard, J. , T. Bengtsson , and B. Cannon . 2007 Unexpected evidence for active brown adipose tissue in adult humans. Am. J. Physiol. Endocrinol. Metab. 293:E444–E452. 17473055 10.1152/ajpendo.00691.2006

[phy213807-bib-0035] Papp, R. S. , and M. Palkovits . 2014 Brainstem projections of neurons located in various subdivisions of the dorsolateral hypothalamic area‐an anterograde tract‐tracing study. Front. Neuroanat. 8:34. 24904303 10.3389/fnana.2014.00034 PMC4032949

[phy213807-bib-0036] Rathner, J. A. , and S. F. Morrison . 2006 Rostral ventromedial periaqueductal gray: a source of inhibition of the sympathetic outflow to brown adipose tissue. Brain Res. 1077:99–107. 16499889 10.1016/j.brainres.2006.01.035

[phy213807-bib-0037] Rezai‐Zadeh, K. , and H. Munzberg . 2013 Integration of sensory information via central thermoregulatory leptin targets. Physiol. Behav. 121:49–55. 23458626 10.1016/j.physbeh.2013.02.014 PMC3683124

[phy213807-bib-0038] Rezai‐Zadeh, K. , S. Yu , Y. Jiang , A. Laque , C. Schwartzenburg , C. D. Morrison , et al. 2014 Leptin receptor neurons in the dorsomedial hypothalamus are key regulators of energy expenditure and body weight, but not food intake. Mol. Metab. 3:681–693. 25352997 10.1016/j.molmet.2014.07.008 PMC4209380

[phy213807-bib-0039] Rosenbaum, M. , and R. L. Leibel . 2010 Adaptive thermogenesis in humans. Int. J. Obes. 34(Suppl 1):S47–S55. 10.1038/ijo.2010.184 PMC3673773 20935667

[phy213807-bib-0040] Saper, C. B. , L. W. Swanson , and W. M. Cowan . 1976 The efferent connections of the ventromedial nucleus of the hypothalamus of the rat. J. Comp. Neurol. 169:409–442. 61975 10.1002/cne.901690403

[phy213807-bib-0041] Seale, P. , and M. A. Lazar . 2009 Brown fat in humans: turning up the heat on obesity. Diabetes 58:1482–1484. 19564460 10.2337/db09-0622 PMC2699856

[phy213807-bib-0042] da Silva, L. G. , R. C. A. de Menezes , R. A. S. dos Santos , M. J. Campagnole‐Santos , and M. A. P. Fontes . 2003 Role of periaqueductal gray on the cardiovascular response evoked by disinhibition of the dorsomedial hypothalamus. Brain Res. 984:206–214. 12932855 10.1016/s0006-8993(03)03157-3

[phy213807-bib-0043] Thompson, R. H. , N. S. Canteras , and L. W. Swanson . 1996 Organization of projections from the dorsomedial nucleus of the hypothalamus: a PHA‐L study in the rat. J. Comp. Neurol. 376:143–173. 8946289 10.1002/(SICI)1096-9861(19961202)376:1<143::AID-CNE9>3.0.CO;2-3

[phy213807-bib-0044] Thornhill, J. , and I. Halvorson . 1994 Activation of shivering and non‐shivering thermogenesis by electrical stimulation of the lateral and medial preoptic areas. Brain Res. 656:367–374. 7820598 10.1016/0006-8993(94)91481-8

[phy213807-bib-0045] Villela, D. C. , L. G. da Silva , Jr Fontes , M. A. . 2009 Activation of 5‐HT receptors in the periaqueductal gray attenuates the tachycardia evoked from dorsomedial hypothalamus. Auton. Neurosci. 148:36–43. 19303372 10.1016/j.autneu.2009.02.004

[phy213807-bib-0046] Virtanen, K. A. , M. E. Lidell , J. Orava , M. Heglind , R. Westergren , T. Niemi , et al. 2009 Functional brown adipose tissue in healthy adults. N. Engl. J. Med. 360:1518–1525. 19357407 10.1056/NEJMoa0808949

[phy213807-bib-0047] Wickersham, I. R. , S. Finke , K. K. Conzelmann , and E. M. Callaway . 2007 Retrograde neuronal tracing with a deletion‐mutant rabies virus. Nat. Methods 4:47–49. 17179932 10.1038/NMETH999 PMC2755236

[phy213807-bib-0048] Yoshida, K. , M. Konishi , K. Nagashima , C. B. Saper , and K. Kanosue . 2005 Fos activation in hypothalamic neurons during cold or warm exposure: projections to periaqueductal gray matter. Neuroscience 133:1039–1046. 15927405 10.1016/j.neuroscience.2005.03.044

[phy213807-bib-0049] Zaretskaia, M. V. , D. V. Zaretsky , A. Shekhar , and J. A. DiMicco . 2002 Chemical stimulation of the dorsomedial hypothalamus evokes non‐shivering thermogenesis in anesthetized rats. Brain Res. 928:113–125. 11844478 10.1016/s0006-8993(01)03369-8

[phy213807-bib-0050] Zhang, Y. , I. A. Kerman , A. Laque , P. Nguyen , M. Faouzi , G. W. Louis , et al. 2011 Leptin‐receptor‐expressing neurons in the dorsomedial hypothalamus and median preoptic area regulate sympathetic brown adipose tissue circuits. J. Neurosci. 31:1873–1884. 21289197 10.1523/JNEUROSCI.3223-10.2011 PMC3069639

